# XRIndex: a brief screening tool for individual differences in security threat detection in x-ray images

**DOI:** 10.3389/fnhum.2015.00439

**Published:** 2015-08-10

**Authors:** Elena Rusconi, Francesca Ferri, Essi Viding, Timothy Mitchener-Nissen

**Affiliations:** ^1^Department of Security and Crime Science, University College LondonLondon, UK; ^2^Department of Neurosciences, University of ParmaParma, Italy; ^3^Division of Psychology, Abertay UniversityDundee, UK; ^4^Institute of Mental Health Research, University of OttawaOttawa, ON, Canada; ^5^Division of Psychology and Language Sciences, University College LondonLondon, UK

**Keywords:** security and human factors, autism spectrum disorders, applied psychology, threat detection, x-ray imaging

## Abstract

X-ray imaging is a cost-effective technique at security checkpoints that typically require the presence of human operators. We have previously shown that self-reported *attention to detail* can predict threat detection performance with small-vehicle x-ray images (Rusconi et al., 2012). Here, we provide evidence for the generality of such a link by having a large sample of naïve participants screen more typical dual-energy x-ray images of hand luggage. The results show that the Attention to Detail score from the autism-spectrum quotient (AQ) questionnaire (Baron-Cohen et al., 2001) is a linear predictor of threat detection accuracy. We then develop and fine-tune a novel self-report scale for security screening: the XRIndex, which improves on the Attention to Detail scale for predictive power and opacity to interpretation. The XRIndex is not redundant with any of the Big Five personality traits. We validate the XRIndex against security x-ray images with an independent sample of untrained participants and suggest that the XRIndex may be a useful aid for the identification of suitable candidates for professional security training with a focus on x-ray threat detection. Further studies are needed to determine whether this can also apply to trained professionals.

## Introduction

When London was chosen to stage the 2012 Olympic and Paralympic Games security was a major concern, with threats ranging from low-level crime to a major terrorist attack. Extra security checkpoints were required, in particular at Olympic venues. Because most checks require the deployment of technology (e.g., x-ray machines, biometrics scanners, metal detectors) and human personnel, machinery had to be acquired and personnel hired and trained on a temporary basis. The security firm G4S was appointed to provide, train and manage security personnel to fulfil roles such as *Level 2 Award* accredited Door Supervisors, Bag Searchers and Screeners, and x-ray operators.

According to a Home Affairs Committee, 2012 report (House of Commons, Seventh Report of Session Home Affairs Committee, 2012, p. 2), “G4S was (eventually) contracted to recruit, train and accredit 10,400 staff and manage 13,000 others. The total number of security personnel required for the Games was 23,700”. However, when it became clear that G4S would not be able to deliver the requested numbers 10,700 troops and police officers working overtime had to be deployed on short notice to address the shortcoming (much of it attributed by government to G4S’s poor practices in handling the candidates). The costs associated with the last-minute recruitment and overtime work of security professionals might have been partly avoided if tools enabling a fast and agile assessment for the recruitment and training of large numbers of *ad hoc* security personnel had been available.

Security x-ray image interpretation is a typical skill that security personnel may need to acquire for working at large scale events, in aviation security, at the entry points of courts and penal facilities, and elsewhere. In a security context, x-ray image interpretation is aimed at the identification of potential threats and/or illicit material in bags or vehicles. It requires an eye for detail and the ability to distinguish potentially harmful items from non-harmful ones. It also presupposes fluent knowledge of illicit objects and materials, familiarity with their appearance (hence the importance of specific training), and of the operating procedures connected with the detection of potential threats (see e.g., Schwaninger, 2006, for a cognitive task analysis). In standard working conditions, this is by no means the only task in which a security officer will be deployed, if for no other reason than the physiological limitations of sustaining attention which require shifts lasting only 20 min. However, the sheer volume of bags requiring screening every day at security checkpoints is such that developing interventions aimed at improving screening effectiveness deserves to be prioritized. These can seek to improve the available technology, strengthen the training provided to security personnel or develop *ad hoc* assessment and evaluation tools to help optimize the match between individual potential and job requirements.

Schwaninger et al. (2005) and Hardmeier et al. (2005) developed and validated a test of x-ray screening abilities, the X-Ray Object Recognition Test (ORT), that requires minimal background knowledge and simulates the security x-ray screening task. Their studies highlighted a series of image characteristics that make the object recognition task more challenging for the human visual system in an x-ray screening context than with more naturally occurring images. In typical x-ray images, target objects are likely to present themselves from unusual views, often with superposed objects and within a complex clutter. Although task-specific training can greatly improve performance (Koller et al., 2008, 2009), large individual differences in coping with such image-based factors persist, as shown by studies in which novices and trained professionals are tested with the same protocol (Hardmeier et al., 2005; Schwaninger et al., 2005, 2010; Hardmeier and Schwaninger, 2008). It has also been shown that some of the weakest trained screeners may perform worse than the best untrained screeners (Halbherr et al., 2013).

This suggests that, alongside the development of professional training routines, the development of tools that help assess an individual’s predisposition to x-ray image interpretation could play a crucial role in improving screening effectiveness. Such tools may include: carefully designed and representative job samples like the X-Ray ORT; tests like Raven’s Advanced Progressive Matrices (Raven et al., 1980; e.g., used in Hardmeier and Schwaninger, 2008); or the Attention to Detail scale (Baron-Cohen et al., 2001; e.g., used in Rusconi et al., 2012) developed for a range of general purposes.

Ability tests offer the obvious advantage of capturing differences in samples of individual performance, but require access to specialized material and are relatively time consuming. Self-report tests are very popular in personnel selection and offer the advantage of being easy and quick to administer to the general population, but are heavily reliant on individuals’ insight and honesty. Lack of insight may be detected by analyzing an individual’s behavioral performance or by collecting external observers’ reports, whereas lack of honesty may be often detected in the response pattern emerging from well-designed self-report instruments.

Significant correlations have been reported between individual ratings of x-ray image interpretation difficulty based on unusual views, superposition and complexity, and objective measures of image interpretation difficulty as shown by threat detection performance (Schwaninger et al., 2007). It is reasonable to expect that some of the visual-analytic skills required for x-ray image interpretation and that, likely, also shape an individual’s perceptual experience of the visual world may be accessible to self-awareness.

In a previous study, we suggested that individuals who spontaneously focus on visual detail may be well-suited to identify target objects in security x-ray images (Rusconi et al., 2012). We used a self-report measure of *attention to detail* as found in the autism-spectrum quotient (AQ) questionnaire (Baron-Cohen et al., 2001). Indeed, high levels of piecemeal attention are typically present in autism spectrum disorders (ASD), clustering together with restricted interests, poor imagination, social and communication impairments. This hyperattention to detail may associate with better-than-average performance in certain behavioral tasks, such as the Embedded Figures Test and the Corsi Block Test (e.g., Shah and Frith, 1983; Happé et al., 2001), implying superior search and/or identification of visual objects in a complex image. It is possible to identify individuals with high and low attention to detail in the general population, with ASD individuals located at the higher end of the trait continuum (Baron-Cohen et al., 2001). Whereas the diagnosis of ASD is always based on the concomitant presence of social and communication impairments—although their relative weight may vary widely between individuals—in the general population high attention to detail does not necessarily associate with other ASD traits (see e.g., Rusconi et al., 2012). This is fully consistent with the view of ASD as a fractionable constellation of symptoms (Happé et al., 2006), according to which every ASD defining trait is the expression of a relatively independent set of genes. When all traits appear concomitantly, they give rise to a clinically relevant entity.

In the current Study 1, we probed the generality of the connection between self-reported attention to detail and behavioral differences in an x-ray screening task. To this aim we used dual-energy x-ray images of hand luggage and tested a much larger sample of participants than in our previous study. This allowed us to probe a range of scores along the Attention to Detail scale against typical security images, rather than just extreme scores. Having ascertained the validity of Attention to Detail as a predictor for screening performance, we proceeded to identify the most predictive items within the original self-rating scale. A more targeted instrument was then generated; namely the XRIndex building on the Attention to Detail scale. In Study 2, we validated the XRIndex with an independent sample of participants and showed that the trait it measures is not redundant with any of the Big Five personality traits. Because the core sample of participants who contributed to Study 1 were recruited in Italy, whereas the core sample of participants who contributed to Study 2 were recruited in the UK, this also probed the cross-cultural validity of the XRIndex. In Study 3 we retested a subgroup of participants who took part in Study 2 and provided test-retest reliability measures. We end by suggesting the utility of the XRIndex for the identification of individuals with a predisposition to the x-ray screening task. Further validation studies are in the making to test whether the approach could be extended to the selection of trained professionals.

## Study 1

### From Attention to Detail to the XRIndex: Fine-Tuning and Preliminary Validation

#### Materials and Methods

##### Participants

Participants were recruited via departmental mailing lists and personal contacts at University of Parma and University of Pescara and Chieti (Italy). The core sample consisted of 215 participants (53 males) with a complete dataset (i.e., AQ questionnaire, Phase 1 and Phase 2 tests; see below), although larger samples with partial datasets (max *N* = 391) were also available (see below). All of them were naïve as to the purpose of the study. They were aged on average 23 years (SD = 5) and had spent 17 years (SD = 3) in education. Participants who did not have normal vision were requested to wear corrective eyeglasses or contact lenses before taking the tests.

Recruitment occurred as follows: most participants received a first email with a link to the online version of the AQ questionnaire. Between 6 and 9 months later, those who had agreed to volunteer for a successive phase of the study were asked to complete Phase 1 (including the Attention to Detail subscale of the AQ questionnaire, the Ten-Item Personality Inventory—TIPI, and 20 novel items), and those who volunteered for a further phase were asked to complete Phase 2. Some participants dropped out after taking the AQ questionnaire while others were freshly recruited for Phase 1. Partial datasets were used for exploratory analyses and scale development purposes which included participants who completed both the AQ questionnaire and Phase 1 (283 participants: 65 males; age: *M* = 23, SD = 5; education: *M* = 16, SD = 3) and participants who completed Phase 1 only (338 participants: 118 males; age: *M* = 23, SD = 5; education: *M* = 16, SD = 3). For the AQ questionnaire, data from 391 participants were available and these, in conjunction with data from external samples (i.e., the sample of Rusconi et al., 2012, *N* = 124, and an additional small sample from the same population, *N* = 32, recruited after completion of that study), have been used for the Exploratory Factor Analysis (EFA), thus reaching a total *N* of 547. Note that the AQ questionnaire has been cross-culturally validated (see Autism Research Centre website, and Ruta et al., 2012) therefore we can expect the English and Italian versions to present the same factorial structure.

##### Stimuli

The overall testing protocol included a novel self-report scale in preliminary form, a series of validated questionnaires and an x-ray screening task. Additional tasks were also included in the testing protocol but they were not aimed to scale validation and will thus be the object of a separate report.

###### Autism-spectrum quotient (AQ) Questionnaire and Attention to Detail Subscale

The AQ questionnaire was developed and validated by Baron-Cohen et al. (2001) to test ASD-related traits in the general population. It includes five subscales (Attention to Detail, Attention Switching, Imagination, Communication Skills, Social Skills) tapping different aspects of ASD with 10 items each. Respondents were to rate each item (e.g., “I often notice small sounds when others do not”) on a 4-level Likert scale (Definitely agree, Slightly agree, Slightly disagree, Definitely disagree). The Attention to Detail subscale was made of items 5, 6, 9, 12, 19, 23, 28, 29, 30, 49 from the AQ questionnaire.

###### Ten-Item Personality Inventory (TIPI)

The TIPI was developed and validated by Gosling et al. (2003) as a brief inventory for the Big Five personality traits whereby respondents were requested to rate 10 pairs of descriptive items such as “Disorganized, careless” on a 7-level Likert scale (1 = Disagree strongly, 7 = Agree strongly).

###### Novel scale: proto-XRIndex

The proto-XRIndex contained 20 items conceptually derived from the Attention to Detail scale, focusing on those aspects that may be more relevant to the x-ray screening task (see also “Zooming in on the Attention to Detail scale (*N* = 547)” in “Results” Section). It was formulated in such a way that they did not always require positive responses and required careful reading. Like in the Attention to Detail scale, respondents were to rate each item on a 4-level Likert scale (Definitely agree, Slightly agree, Slightly disagree, Definitely disagree).

###### X-ray screening task

The x-ray screening task was partly modeled on Rusconi et al.’s (2012) protocol but used color-coded x-ray images of hand luggage. Color-coding is an enhancement function available on airport security x-ray machines. X-ray machines do not produce colored images but density maps. However, when projecting density maps onto visual displays, it is possible to automatically assign different colors to different density ranges indicating different materials (e.g., blue for metal, orange for biological, green for alloys). Images of a set of bags with and without threats were created by colleagues from the Home Office Centre for Applied Science and Technology (CAST) in Sandridge (UK). They were generated at optimal irradiation parameters as determined by the x-ray machine builder with an x-ray machine implementing state-of-the-art technology. The machine was being tested by CAST scientists for mass adoption at major checkpoints in the London 2012 Olympics.

**Figure 1:**
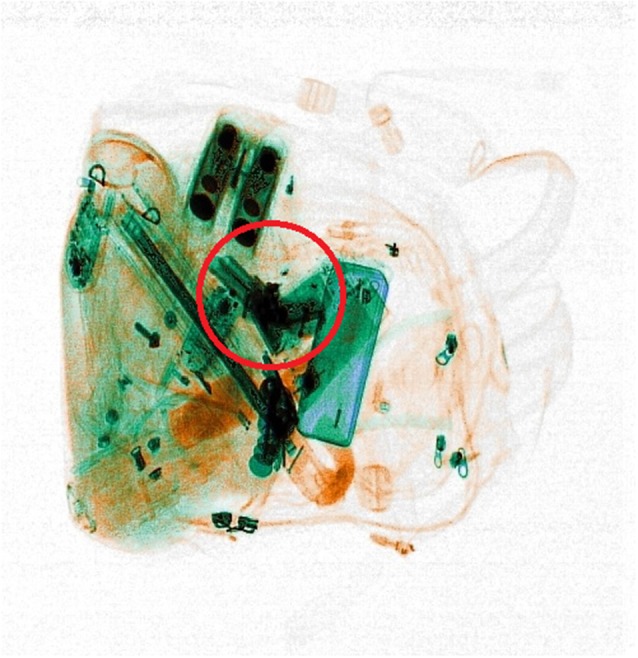
**Example of the x-ray images used in the x-ray screening task**. The red circle locates the threat for demonstrative purposes. It was shown in the feedback displays of the practice trials but did not appear in the experiment.

From an initial database of 100 images, 15 pairs were selected. Each pair showed the same bag with/without a threat (see an example in Figure 1). A further 15 pairs were obtained by mirroring those images around their vertical and then their horizontal meridian. This introduced a balance in the spatial location of visual clusters, by ensuring that in the overall set of 60 images (i.e., 30 pairs of threat/no-threat bags) a threat—and any other object—appeared with the same likelihood in the opposite halves with respect to the vertical and the horizontal meridians. During the x-ray screening task, the images—which had a white background—were inscribed in a 600 × 600 pixel central square, whereas the rest of the display was black.

##### Procedure

###### Testing mode

Participants were tested online and remotely (henceforth: *online testing* indicates the use of questionnaires on a webpage; *remote testing* indicates the use of dedicated software downloaded from a website and installed on their own computer). A testing session consisted of two main phases: the first comprising only online questionnaires; the second and more demanding phase including both questionnaires and behavioral tasks administered via dedicated software. When participants were tested remotely the software was to be downloaded and installed directly by every participant on their PC.

###### Phase 1: Web questionnaires

Phase 1 was accessible via open web-link and comprised: the 10-item Attention to Detail subscale from the AQ questionnaire (Baron-Cohen et al., 2001), the TIPI (Gosling et al., 2003) and a 20-item pilot version of a novel self-report scale (the proto-XRIndex). Note that most individuals who completed the first version had already completed the AQ questionnaire online a few months earlier. The online platform used to test participants was LimeSurvey™.

The questionnaires were preceded by an informed consent page, which referred to the study as being generically focused on visuospatial abilities so as not to give away the rationale of the research. If participants gave their consent and confirmed they were at least 18 years of age a page followed requesting to input personal information (e.g., the University/Company affiliation, phone number and city of residence for the Italian version of the battery). This was partly relevant for the study, partly aimed to discourage/detect multiple data submissions from the same individual. Demographic information about age and education was also to be provided. Finally, participants were requested to input their email address, enabling the experimenter to send them personalized instructions for Phase 2. Additional information was requested regarding degrees, qualifications and work experience. This was intended to help detect and exclude from the sample of naïve participants any individuals with previous experience or qualifications in the security sector (a more explicit question was also included in the remote testing battery). Once all the fields were filled, pressing the “Next” button brought participants onto the following page where the first of three questionnaires was to be completed.

The questionnaires were administered in two orders starting either with the Attention to Detail subscale or with the proto-XRIndex, with the Attention to Detail subscale and the proto-XRIndex always separated by the TIPI to avoid mechanical responses to items that may have been perceived as insisting on very similar concepts. Like in the original versions of the questionnaires, participants could select only one of the available responses for each item and were encouraged not to skip any items, as missing responses would have compromised the validity of the questionnaires. In fact, they could not move on to the next page until they had answered all items of the current page. On completion of the third questionnaire, pressing the “Next” button would make the final page appear. With it, participants were given the possibility to leave any comments or feedback they may have on the testing session. Finally they were required to select one of two options: either agreeing to proceed with the testing (and thus to be contacted via email with further instructions) or deciding to withdraw from the study. By clicking on the “Submit” button, participants effectively completed Phase 1 while sending information about their availability for further testing. If a participant agreed to volunteer for the following phase, s/he would then receive an email message with instructions and personal codes for accessing the following testing protocol.

###### Phase 2: X-ray screening task

After completion of Phase 1 and consenting to take part in Phase 2, participants received a personalized email with detailed instructions on how to complete Phase 2. In order to access the computer tasks participants were advised they would need a PC with Internet connection. They were directed to a web page from where they could safely download and install the dedicated testing software, Psytools (Delosis), which provided a stable testing environment and ensured that data were collected and retrieved securely from a central server. If participants had any concerns or experienced any problems during the installation, they were encouraged to contact the research team for assistance.

With their personalized email participants also received three unique codes: a Player Code, a Player Key and a Password. The Player Code and Key were necessary to enable the installation, task loading and data logging, and the Password was necessary to gain access to the tasks on later occasions. In case participants had installed the software on shared computers and did not complete all the tasks in one go, this prevented other users from accessing the incomplete tasks by simply launching Psytools with a double-click on its Desktop shortcut icon. Installing and loading the tasks from the Delosis homepage required internet connection for just a few seconds to communicate with the Delosis computers. However, there was no need to be on-line to run the tasks, and participants were informed about this. They also learnt that the programme would send the results back to Delosis over the Internet when they had completed the tasks, taking just a few seconds. All the information that participants sent over the Internet was kept strictly confidential and anonymous. Moreover, personal data, e.g., name and email address, which were provided in Phase 1 for the purpose of identification and to allow the researchers to contact each respondent individually were kept separate from the data collected in Phase 2.

On successfully installing the software and entering the user window, a list of tasks, including the x-ray screening task, would appear on a participant’s PC screen, ready to be run. The instruction letter emphasized the importance of answering all of the tasks on one’s own. Crucially, a task could be started once and only once. This was aimed to prevent participants from taking a first look, exit, restart, exit, and so forth until they became familiar with the task before deciding to complete the entire session. They were thus also made aware that they should start a task only when they knew that nobody would interrupt them and were asked to switch their mobile phone off while completing the testing battery. Finally, instructions mentioned that accuracy and speed of response would be recorded and participants were encouraged to be as fast and accurate as possible. Data were logged on the system and automatically sent to the Psytools central server (therefore being accessible to the experimenter) as soon as an internet connection became available on each participant’s computer.

**Figure 2:**
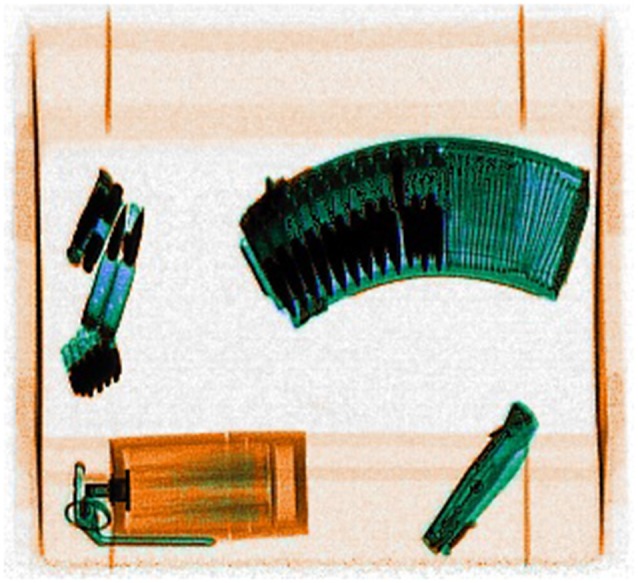
**Example threat display presented in the familiarization phase of the x-ray screening task**.

The x-ray screening task comprised two phases: a familiarization phase and a threat detection phase. The threat detection phase entailed a few training trials, followed by test trials. When accessing the x-ray screening task participants were advised they would first be shown images of three groups of threats (see e.g., Figure 2). They were encouraged to try and memorize those threats because they would later be asked to detect their presence in x-rayed bags. It was also made clear that the threats would not necessarily be seen from the same viewpoint as in the familiarization phase. Images showing three collections of x-rayed threats (bullets, a gas grenade and a folding knife; several handguns; different types of knives) were displayed twice for 6 s each in alternated sequence. Six threat-detection practice trials (three bags with a threat, three bags without, in random order) followed the familiarization phase.

For the practice trials and during the entire experiment, participants were instructed to place their right index finger on the P key, their left index finger on the Q key and their thumbs on the spacebar. They were to indicate whether the x-rayed bags contained a threat or not by pressing P as quickly as possible if they had spotted a threat, by pressing Q if they thought there was no threat. Each image would remain on the screen for a maximum time of 6 s, which was also the deadline for response. In the practice trials, feedback would be provided immediately after a response (or when the maximum available time had elapsed) and threats, if any, highlighted with a red circle on the image (see e.g., Figure 1). Participants could take all the time they wanted to inspect the feedback screen and the next trial appeared only when they pressed the spacebar.

After completing the six practice trials and receiving feedback (response accuracy, response time and location of the threat) for each, participants started the actual experiment. The experiment followed the same trial structure as the practice phase, but the feedback in this case was limited to a green V for a correct response and a red X for a wrong response. Cumulative accuracy and average reaction time (RT) were also shown on the same display as the feedback after each trial. When ready to move on to the next trial participants pressed the spacebar and a new x-rayed bag would appear. They were informed by a message on the screen when they had reached the middle of the test. Overall, the task comprised 60 experimental trials and lasted less than 10 min but required participants’ undivided attention throughout.

##### Data analysis

Data from Study 1 were used to test the validity of the Attention to Detail scale as a predictor of x-ray screening performance with typical security images, and to fine-tune an *ad-hoc* self-report scale—the XRIndex. In this and the following studies, questionnaire scores and behavioral performance indices were treated as interval data, in line with most of the literature. Because these data do not always meet all parametric test assumptions, we either performed non-parametric tests or provided bootstrapped estimates and Bias Corrected and accelerated (BCa) 95% confidence intervals (CIs) alongside the most critical parametric tests (Efron and Tibshirani, 1993). All reported significance levels refer to the two-tailed hypothesis. Bonferroni-Holm corrections for multiple tests were applied in exploratory analyses.

A series of Spearman’s correlations was first performed to assess the relative independence of the Attention to Detail traits from other ASD-related traits in healthy adults. Five indices of performance (three of which resulted significantly related to Attention to Detail in Rusconi et al., 2012) were extracted from behavioral data in the x-ray screening task: (i) percentage of accurate responses; (ii) Hits minus False Alarms (HFA), (iii) sensitivity (*d’*); (iv) criterion (*c*); and (v) Reaction Times (RTs). Performance was first explored via ANOVA or *t*-tests having Threat (threat present, threat absent) and Block (first half, second half) as within-participant factors. Linear regressions were then used to test for the validity of Attention to Detail as a predictor of x-ray screening performance. After formulating the hypothesis that the predictive value of the Attention to Detail scale may reside in specific clusters of items, an ordinal EFA was performed on all of the available data collected by this group with the AQ questionnaire (including Rusconi et al.’s 2012). This highlighted the possibility to parcellate the construct into three components, only two of which were related with x-ray screening performance—one positively correlated, one negatively correlated with it. A new predictive index was thus derived from the original Attention to Detail scale by combining the negatively related items with the positively related items. The most strongly associated items from our new pool of items were also included, to create a 10-item scale, which we named the XRIndex. Cronbach’s α was also calculated. A series of hierarchical linear regressions tests was then performed to assess whether the XRIndex can predict performance over and above the Attention to Detail scale.

### Results

#### Attention to Detail Predicts Screening Performance with Dual-Energy X-Ray Images (*N* = 215)

For each AQ subscale, a total score was obtained by counting the number of relevant items for which respondents had selected the ASD-related preference, as in Baron-Cohen et al. (2001). Central tendency statistics for the five AQ subscales and the total AQ score are shown in Table 1. Only two participants slightly exceeded the theoretical threshold of clinical relevance obtaining a total score of 33. Since they reported no clinical history and did not classify as outliers in other measures, they were retained in the final sample. A series of Spearman’s correlations highlighted a significant positive correlation between the Communication subscale and the Social Skill and Attention Switching subscales (ρ = 0.45 and ρ = 0.33 respectively), and between the Social Skill and Attention Switching subscales (ρ = 0.19). Like in Rusconi et al. (2012), no significant correlation was found between the Attention to Detail subscale and the other subscales contributing to the total AQ.

**Table 1:** **Descriptive statistics obtained with the binary scoring of the Autism Quotient (AQ) questionnaire and Spearman’s correlations (*p*-values in Italics) between AQ component scales**.

	Median (IQR)	Soc Skill	Att Switch	AttDet	Comm	Imag
**Soc Skill**	2 (2)
**Att Switch**	4 (3)	0.19* 0.005
**AttDet**	6 (4)	**0.00** ***0.941***	−**0.09** ***0.188***
**Comm**	2 (2)	0.45* 0.000	0.33* 0.000	−**0.04** ***0.515***
**Imag**	2 (2)	0.13 *0.055*	0.06 *0.358*	−**0.10** ***0.124***	0.12 0.068
**TOTAL AQ**	16 (6)	0.62* 0.000	0.55* 0.000	0.37* 0.000	0.65* 0.000	0.37* 0.000

Correlation tests involving the Attention to Detail scale are highlighted with bold font. Soc Skill, Social Skill scale of the AQ questionnaire; Att Switch, Attention Switching scale; AttDett, Attention to Detail scale; Comm, Communication scale; Imag, Imagination scale.*significant with Bonferroni-Holm correction (min corrected α = 0.003), N = 215.

The main dependent variables considered for the x-ray screening task were: *accuracy* (% total trials)*, hits minus false alarms* or HFA (%), *sensitivity* [d’ = Z (hit rate)−Z (false alarm rate)], *criterion* [*c* = −0.5 (Z (hit rate) + Z (false alarm rate))] and *reaction times* (RTs, measured in milliseconds). The first three of these were shown to be significantly related to Attention to Detail by Rusconi et al. (2012), whereas no difference was found in RTs between groups. Two main independent variables (within participants) were included in the exploratory analysis when possible: Block (1 vs. 2) and Threat (present vs. absent).

- *Accuracy*: On average, participants responded correctly 77% (SE = 0.50) of the times and were more accurate in the presence than in the absence of a threat (*M* = 78%, SE = 0.60 and *M* = 75%, SE = 0.60 respectively, *F*_(1,214)_ = 12.75, *p* < 0.001, partial η^2^ = 0.06). Accuracy increased with practice (Block 1: *M* = 75%, SE = 0.60; Block 2: *M* = 79%, SE = 0.60; *F*_(1,214)_ = 34.86, *p* < 0.001, partial η^2^ = 0.14). No other significant effects were found.
- *HFA*: On average, participants showed an HFA of 54% (SE = 1.00), which increased with practice (Block 1: *M* = 50%, SE = 1.20; Block 2: *M* = 58%, SE = 1.20; *F*_(1,214)_ = 30.53, *p* < 0.001, partial η^2^ = 0.12).
- *Sensitivity*: Participants showed an average d’ of 1.42 (SE = 0.03). Sensitivity improved with practice too (Block 1: *M* = 1.35, SE = 0.04; Block 2: *M* = 1.50, SE = 0.05; *F*_(1,214)_ = 6.05, *p* = 0.015, partial η^2^ = 0.03).
- *Criterion*: Participants showed an average c of −0.01 (SE = 0.02). Response criterion did not change with practice (Block 1: *M* = −0.01, SE = 0.02; Block 2: *M* = −0.02, SE = 0.02; *F* < 1).
- *Reaction Times*: On average, participants responded correctly in 1667 ms (SE = 28) and were faster in the presence than in the absence of a threat (*M* = 1296 ms, SE = 19 and *M* = 2037 ms, SE = 24 respectively, *F*_(1,214)_ = 511.68, *p* < 0.001, partial η^2^ = 0.70). Speed increased with practice (Block 1: *M* = 1769 ms, SE = 33; Block 2: *M* = 1565 ms, SE = 27; *F*_(1,214)_ = 81.39, *p* < 0.001, partial η^2^ = 0.28) and more so for threat absent than threat present items (Threat × Block: *F*_(1,214)_ = 10.46, *p* = 0.001, partial η^2^ = 0.05).

For direct comparability with Rusconi et al.’s (2012) study, participants obtaining a score lower than four (i.e., participants whose score fell in the lower quartile of the distribution), or higher than eight (i.e., participants whose score fell in the upper quartile of the distribution) in the Attention to Detail subscale were assigned to two independent groups: Low Attention to Detail (*N* = 34, 28 females) and High Attention to Detail (*N* = 26, 18 females). Independent samples *t*-tests revealed a significant difference between groups for accuracy (all trials: *t*_(58)_ = 2.71, *p* = 0.009; threat present trials: *t*_(58)_ = 3.10, *p* = 0.003; threat absent trials: *t*_(58)_ = 1.12, *p* = 0.268), HFA (*t*_(58)_ = 2.74, *p* = 0.008), d’ (*t*_(58)_ = 2.87, *p* = 0.006), but not RTs (all trials: *t*_(58)_ = 0.36, *p* = 0.719; threat present trials: *t*_(58)_ = 1.50, *p* = 0.139; threat absent trials: *t*_(58)_ = −0.23, *p* = 0.820) or c (*t*_(58)_ = 1.09, *p* = 0.317) in the x-ray screening task. In all cases where a significant difference was found, the High Attention to Detail group outperformed the Low Attention to Detail group (Mean difference between High and Low for Accuracy = 5%, threat present = 7%; HFA = 11%; d’ = 0.41; see also Table 2). Given the larger sample available for this study, linear regression models having Attention to Detail as predictor were also tested for all the indices of performance. These revealed a significant linear trend in threat detection accuracy (*R*^2^ = 0.04, constant = 74, *b* = 0.74, *p* = 0.003; see also Table 2) and linear trends in HFA and d’ approaching significance (HFA: *R^2^* = 0.02, constant = 49, *b* = 0.81, *p* = 0.070; d’: *R^2^* = 0.02, constant = 1.37, *b* = 0.03, *p* = 0.058). No significant effect was found with c and RTs (*p* = 0.173 and *p* = 0.940 respectively). In the following section we will try to identify whether the predictive value of the Attention to Detail scale may reside in specific clusters of items.

**Table 2:** **Robust statistics, Bias Corrected and accelerated (BCa) 95% confidence intervals (CIs) and significance levels for indices of performance with crucial significant parametric tests in Study 1, Study 2 and Study 3**.

Index of performance	Statistic	BCa 95% CI	Significance level (*p*)
**STUDY 1**
	***Mean difference***
	*High – Low Attention to Detail group*		
Accuracy	5.34	1.71 – 8.92	0.012
Accuracy (threat)	7.22	2.61 – 11.78	0.005
HFA	10.90	3.52 – 18.26	0.010
d’	0.41	0.14 – 0.68	0.010
	***Regression coefficient b** for Attention to Detail*
Accuracy (threat)	0.74	0.24 – 1.22	0.003
	***Regression coefficient b** for XRIndex*
Accuracy	0.44	0.21 – 0.67	0.001
Accuracy (threat)	0.33	0.06 – 0.64	0.025
Accuracy (no threat)	0.54	0.18 – 0.89	0.004
HFA	0.87	0.39 – 1.34	0.001
d’	0.03	0.02 – 0.05	0.001
**STUDY 2**			
	***Regression coefficient b** for XRIndex*
Accuracy	0.56	0.33 – 0.78	0.001
Accuracy (threat)	0.43	0.15 – 0.67	0.005
Accuracy (no threat)	0.70	0.36 – 1.04	0.001
HFA	0.86	0.37 – 1.37	0.001
d’	0.03	0.01 – 0.05	0.006
**STUDY 3**			
	***Regression coefficient b** for XRIndex*
Accuracy	0.56	0.17 – 0.95	0.009
Accuracy (threat)	0.55	0.19 – 0.93	0.010
HFA	1.11	0.42 – 1.89	0.004
d’	0.05	0.01 – 0.07	0.004

*Estimates are based on 1000 bootstrap samples*.

#### Fine-Tuning Self-Reports on X-Ray Screening Performance

##### Zooming in on the attention to detail scale (*N* = 547)

On closer inspection, the Attention to Detail scale from the AQ questionnaire contains items that cover a range of contents and aspects of piecemeal attention that may not all be relevant for security x-ray screening. In particular, three subgroups of items could be identified based on their textual content: a subgroup concerning single details (e.g., “I tend to notice details that others do not”; AQ items 5, 12, 28 and 30), a subgroup concerning clusters of information (e.g., numbers, patterns and dates; “I usually notice car number plates or similar strings of information”; AQ items 6, 9, 19 and 23), and a subgroup concerning memory (e.g., “I am not very good at remembering people’s date of birth” (reverse scored item); AQ items 29 and 49). So, although the most parsimonious factorial model of Attention to Detail may be unidimensional (Baron-Cohen et al., 2001), a higher-resolution model may be better suited to our aims. Indeed, the ability to identify objects in a cluttered image may rest, for example, more on the ability to connect details of information into a meaningful pattern rather than purely in a sharp focus on single details, as that would distract away from meaningful wholes within the bigger picture.

To retain as much information as possible here, we used 4-level scoring of the AQ items (Hoekstra et al., 2008), whereas binary scoring was used in the previous section to allow for a direct comparison with Rusconi et al.’s (2012) study. With a 4-level coding, however, scale-performance relations did not substantially differ from those obtained with the original binary scoring. We performed an ordinal 3-factor EFA with Full Information Maximum Likelihood extraction method and Promax rotation with LISREL 8.8. A factor was retained if at least two items showed their highest loading under that factor and such loading was larger than 0.45. Ambiguous items showing a difference smaller than 0.30 between their highest and second highest loading were pruned. These criteria complied with the guidelines provided by Field (2009) and Stevens (2002) and enabled us to identify a clear-cut model to guide further research. Each of the three factors comprised two high-loading items and all of the factors were thus retained. Two ambiguous items and two low-loading items were discarded (AQ items 5, 6, 9 and 28), leaving a final selection of six items (AQ items 12, 19, 23, 29, 30 and 49). Factor interpretation was straightforward:

- Factor 1 comprised the items “I tend to notice details that others do not” and “I don’t usually notice small changes in a situation, or a person’s appearance”, and was named *Details*;
- Factor 2 comprised the items “I am fascinated by numbers” and “I notice patterns in things all the time”, and was named *Regularities* (indeed both patterns and numbers are regular systems of elements);
- Factor 3 comprised the items “I am not very good at remembering phone numbers” and “I am not very good at remembering people’s date of birth” was named *Memory*.

Small to moderate correlations were found between factors: Factor 1 and Factor 2: ρ = 0.20; Factor 1 and Factor 3: ρ = 0.19; Factor 2 and Factor 3: ρ = 0.35. We then proceeded to identify the factors or combination of factors that were more strongly associated with x-ray screening performance.

###### Identifying predictive sources in the attention to detail scale (*N* = 215)

In Table 3a, we report the results of Spearman’s correlation analyses between each factor score, derived scores and the relevant indices of performance in the x-ray screening task. The factor *Detail* showed no relation with performance in threat detection. *Regularities* was positively correlated with performance, whereas *Memory* tended to be negatively correlated with performance in the threat detection task before correction for multiple tests. An index derived by subtracting the *Memory* from the *Regularities* scores was positively correlated with most indexes of performance, and remained so after correction for multiple tests.

**Table 3:** **Spearman’s correlations (*N* = 215) between indices of performance in x-ray screening and several combinations of items contained in the Attention to Detail (AttDet) subscale (first (a) and second (b) administration)**.

Index of performance	AttDet Original	Det	Reg	Mem	Det + Reg – Mem	Reg – Mem
**(a) First administration of Attention to Detail scale (in the AQ questionnaire)**
Accuracy	0.10	0.01	0.20	−0.12	0.21	0.24*
	*0.130*	*0.961*	*0.003*	*0.088*	0.21	*0.000*
Accuracy (THREAT)	0.18	0.02	0.19	−0.01	0.10	0.13
	*0.008*	*0.781*	*0.006*	*0.911*	*0.126*	*0.052*
Accuracy (NO THREAT)	−0.01	−0.01	0.12	−0.17	0.21*	0.24*
	*0.989*	*0.889*	*0.081*	*0.015*	*0.002*	*0.000*
HFA	0.11	0.02	0.19	−0.12	0.21*	0.24*
	*0.123*	*0.757*	*0.005*	*0.080*	*0.002*	*0.000*
d’	0.12	0.02	0.19	−0.12	0.21*	0.24*
	*0.099*	*0.795*	*0.005*	*0.084*	*0.002*	*0.000*
**(b) Second administration (~9 months later) of Attention to Detail scale**
Accuracy	−0.07	0.02	0.20	−0.12	0.21*	0.25*
	*0.338*	*0.733*	*0.004*	*0.083*	*0.002*	*0.000*
Accuracy (THREAT)	0.16	0.08	0.22*	−0.01	0.19	0.18
	*0.017*	*0.212*	*0.001*	*0.924*	*0.005*	*0.007*
Accuracy (NO THREAT)	−0.12	−0.03	0.08	−0.16	0.13	0.18*
	*0.080*	*0.889*	*0.256*	*0.016*	*0.056*	*0.007*
HFA	0.01	0.04	0.19	−0.13	0.22*	0.25*
	*0.924*	*0.551*	*0.005*	*0.066*	*0.001*	*0.000*
d’	0.01	0.04	0.20	−0.13	0.22*	0.25*
	*0.953*	*0.553*	*0.004*	*0.055*	*0.001*	*0.000*

The original Attention to Detail (AttDet Original) scale scores are also shown for comparison purposes. Det, Detail; Reg, Regularities; Mem, Memory items of the Attention subscale as identified in the EFA.*significant with Bonferroni–Holm correction (min corrected α = 0.002).

So far we have explored the relation between x-ray screening performance and the Attention to Detail score obtained in the context of the AQ questionnaire. However, the same participants were also administered the Attention to Detail subscale in isolation a few months later. We can thus test for repetition and criterion validity of the *Regularities*−*Memory* subgroup previously identified. Repetition validity was tested by calculating Spearman’s correlation coefficient between the score obtained at the first administration and the score obtained a few months later. Note that this is likely to underestimate the repetition validity coefficient, due to the change in presentation context between test (Attention to Detail items presented within the AQ questionnaire) and retest (Attention to Detail items presented in isolation). The test-retest correlation for *Regularities*−*Memory* was ρ = 0.65, whereas the test-retest correlation for the Attention to Detail subscale (all with four-level scoring) was ρ = 0.75. This is not unexpected as the Attention to Detail score is based on 10 items, whereas the *Regularities*−*Memory* score is based on four items only and may thus be more volatile.

Criterion validity was also retested by correlating indices of performance in threat detection and the Detail, Regularities−Memory items from the Attention to Detail scale administered in isolation a few months after its first administration within the AQ questionnaire (see Table 3b). This supported our previous conclusions, by showing that the *Regularities*−*Memory* score had stronger association than the Attention to Detail score with behavioral performance and were significantly correlated with accuracy, HFA and d’ in the x-ray screening test. However, the small number of items used to calculate the *Regularities*−*Memory* score makes it a potentially volatile index whose reliability would be difficult to assess. We will address this problem in the following section.

#### Towards a Novel Scale: the XRIndex (*N* = 283, *N* = 338 and *N* = 215)

In addition to the Attention to Detail subscale, participants also responded to a pool of 20 novel items in Phase 1. The aim of the following analysis is to enable the identification of those new items that could add to the Attention to Detail items which were identified as best predictors of x-ray screening performance (i.e., the mini-scales *Regularities* and *Memory* comprising two items each). This should help improve (or maintain) the strength and reliability of association between those selected self-report items and x-ray screening performance. At the same time enabling the inclusion of a sufficient number of items in both *Regularities* and *Memory* for calculation of scale reliability indices (e.g., Cronbach’s alpha).

Two-hundred and eighty-three participants (*N* = 283; 65 males; age: *M* = 23, SD = 5; education: *M* = 16, SD = 3) responded to: (1) the AQ questionnaire; (2) the Attention to Detail scale; (3) the new items, and their data and were included in a correlational analysis. Both the new items and the Attention to Detail items from Phase 1 could be correlated with the best predictor of threat detection performance (i.e., *Regularities*−*Memory*) previously identified. The five items (including two Attention to Detail items) that were most positively related and the five items (including two Attention to Detail items) that were most negatively related with the original *Regularities*−*Memory* score were retained as part of the novel scale. A novel index, the XRIndex, was then obtained with the following formula, with both *Regularities* and *Memory* now including two Attention to Detail items and three new items each, and having a similar number of reverse-coded items and negative/positive sentences: *XRIndex* = *Regularities*−*Memory*.

**Figure 3:**
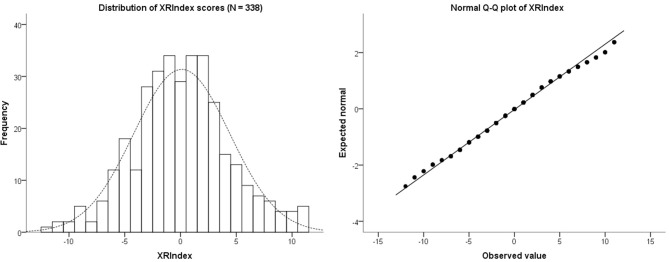
**The left hand panel shows a histogram of the distribution of scores for the XRIndex in 338 participants**. The right hand panel shows the Normal Q-Q plot of the XRIndex distribution, which in its central part essentially overlaps with the normal distribution.

To explore the distribution of the XRIndex scores, we used the entire dataset of 338 participants (118 males) who completed Phase 1—and who had thus responded to both the Attention to Detail scale and the new items. The median XRIndex score was 0 (IQR = 6), and the mean was 0.11 (SD = 4). Skewness and Kurtosis of the XRIndex distribution were very close to 0 (Skewness = 0.10, SE = 0.13; Kurtosis = 0.18, SE = 0.26) and the Q-Q plot showed a reasonable fit of the data with the ideal normal distribution, especially for the central scores (Figure 3). Cronbach’s α was 0.67, which is well within the typical values reported in the social sciences in general and for the AQ subscales in particular (Baron-Cohen et al., 2001; Field, 2009).

**Figure 4:**
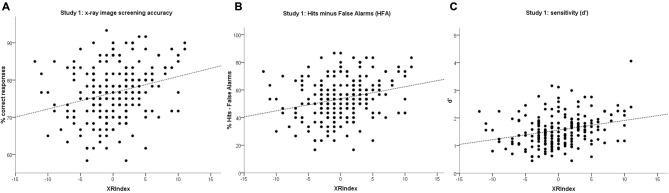
**Scatterplots with linear regression models for the XRIndex on: (A) overall accuracy; (B) Hits minus False Alarms (HFA); and (C) sensitivity (d’) in Study 1**. Each point may represent one or more participants.

We then conducted a preliminary criterion validity check and tested the prediction that the XRIndex would perform better than the Attention to Detail scale at predicting threat detection performance, thanks to the relation of its items with the original “*Regularities* minus *Memory*” index. To do that we used the data from 215 participants for whom both threat detection performance and XRIndex scores were available. Linear regression models having the XRIndex as predictor revealed significant trends in overall detection accuracy (*R^2^* = 0.07, constant = 77, *b* = 0.44, *p* < 0.001), in detection accuracy for threat present items (*R^2^* = 0.03, constant = 78, *b* = 0.33, *p* = 0.009), in rejection accuracy for threat absent items (*R^2^* = 0.05, constant = 74, *b* = 0.54, *p* = 0.001), in HFA (*R^2^* = 0.07, constant = 54, *b* = 0.87, *p* < 0.001), and in d’ (*R^2^* = 0.08, constant = 1.56, *b* = 0.03, *p* < 0.001; see also Table 2; Figure 4) but not in c or RTs (*p* = 0.118 and *p* = 0.922 respectively).

To obtain a simple statistical comparison between Attention to Detail and XRIndex we then tested a series of hierarchical linear regression models on indices of performance in x-ray screening that we had previously related to Attention to Detail. With the exception of detection accuracy, the XRIndex outperformed the Attention to Detail scale as a linear predictor across all indexes of performance. Moreover, the XRIndex was still a significant predictor of performance when considering detection accuracy, whereas the Attention to Detail scale did not significantly predict any of the other performance indexes (see Table 4).

**Table 4:** **Hierarchical regression models having either the XRIndex score or the Attention to Detail (AttDet) score as predictor at Stage 1, and both scores at Stage 2 on all indices of performance**.

Predictor	*R*	*R*^2^	Adj *R*^2^	SE	Δ*R*^2^	Δ*F*	df1	df2	*p*(ΔF)
DV: *Overall accuracy*
**1. XRIndex**	**0.27**	**0.07**	**0.07**	**7**	**0.07**	**16.16**	**1**	**213**	**0.000**
2. XRIndex + AttDet	0.28	0.08	0.07	7	0.01	1.64	1	212	0.201
DV: *Overall accuracy*
1. AttDet	0.12	0.01	0.01	7	0.01	3.18	1	213	0.076
2. AttDet + XRIndex	0.28	0.08	0.07	7	0.06	14.46	1	212	0.000
DV: *Detection Accuracy (threat present items)*
1. XRIndex	0.18	0.03	0.03	8	0.03	7.03	1	213	0.009
2. XRIndex + AttDet	0.25	0.06	0.06	8	0.03	7.27	1	212	0.008
DV: *Detection Accuracy (threat present items)*
**1. AttDet**	**0.20**	**0.04**	**0.04**	**8**	**0.04**	**9.13**	**1**	**213**	**0.003**
2. AttDet + XRIndex	0.25	0.06	0.06	8	0.02	5.20	1	212	0.024
DV: *Rejection accuracy (threat absent items)*
**1. XRIndex**	**0.22**	**0.05**	**0.04**	**11**	**0.05**	**10.81**	**1**	**213**	**0.001**
2. XRIndex + AttDet	0.22	0.05	0.04	11	0.00	0.11	1	212	0.744
DV: *Rejection accuracy (threat absent items)*
1. AttDet	0.01	0.00	0.00	11	0.00	0.02	1	213	0.890
2. AttDet + XRIndex	0.22	0.05	0.04	11	0.05	10.85	1	212	0.001
DV: *HFA*
**1. XRIndex**	**0.26**	**0.07**	**0.06**	**14**	**0.07**	**15.50**	**1**	**213**	**0.001**
2. XRIndex + AttDet	0.27	0.08	0.07	14	0.01	1.76	1	212	0.186
DV: *HFA*
1. AttDet	0.12	0.01	0.01	15	0.01	3.31	1	213	0.070
2. AttDet + XRIndex	0.27	0.07	0.07	14	0.06	13.80	1	212	0.000
DV: *d’*
**1. XRIndex**	**0.27**	**0.07**	**0.07**	**0.54**	**0.07**	**17.19**	**1**	**213**	**0.001**
2. XRIndex + AttDet	0.29	0.08	0.07	0.54	0.01	1.93	1	212	0.166
DV: *d’*
1. AttDet	0.13	0.02	0.01	0.55	0.02	3.62	1	213	0.058
2. AttDet + XRIndex	0.29	0.08	0.07	0.54	0.06	15.34	1	212	0.000

*DV, Dependent Variable; Adj, Adjusted*.

### Discussion

Study 1 provides evidence for the generality of the association between the Attention to Detail trait and threat detection performance with security x-ray images. The association was first reported with a selected sample of participants and single-energy transmission x-ray images of small vehicles (Rusconi et al., 2012). Here we re-tested the original hypothesis with a much larger sample of participants and dual-energy transmission x-ray images of hand baggage.

Taking the Attention to Detail scale as a starting point, a novel self-report scale was then developed, the XRIndex, which could account for up to 7% of the total variance across indices of performance. With a regression coefficient of 0.87 for HFA, performance will increase 0.87 percentage units in HFA for every unit’s increment in XRIndex score. In other words, for every 100 screened bags, an individual obtaining a score of 5 on the XRIndex scale is likely to correctly assess 8.7 more bags than an individual obtaining a score of −5 on the XRIndex. Considering the large volumes of x-ray checks performed worldwide every year (with over 200 million passengers and two million tonnes of freight handled just in the UK; www.gov.uk/dft), the relevance of this finding for aviation security is unmistakable.

## Study 2

### Construct Validation and Extension

#### Materials and Methods

##### Participants

Participants were recruited via departmental mailing lists and personal contacts at University College London and Abertay University (UK). Six-hundred and twenty volunteers (age: *M* = 22 years, SD = 7; education: *M* = 15 years, SD = 3; 232 males) completed Phase 1. Their data were used to test the relation between the XRIndex and personality traits as measured with TIPI. Of these, 165 volunteers (54 males) completed Phase 2 remotely and their data were tested for construct validation of the XRIndex with threat detection, along with data from the first testing session of an additional 108 volunteers (33 males), who completed both Phase 1 and Phase 2 in the laboratory. Because preliminary analyses showed no substantial difference in the pattern of results between the remote sample and the laboratory sample, these datasets were combined to increase statistical power. After removal of far outliers for inverse efficiency measures of performance in behavioral tasks, the final sample comprised 249 participants (i.e., 156 from the remote testing cohort and 93 from the lab testing cohort; 81 males overall). According to the SPSS in-built function, those with a score smaller than Q1−3*IQR or larger than Q3 + 3*IQR are identified as far outliers. The total sample used for the joint analysis was thus of a size comparable with the size of the Italian sample and with similar demographic characteristics (*N* = 249; age: *M* = 22 years, SD = 6, education: *M* = 15 years, SD = 4; 81 males). All of our participants were naïve as to the purpose of the study. Both Phase 1 and Phase 2 instructions explicitly requested participants who did not have normal vision to wear corrective eyeglasses or contact lenses before starting the tests.

##### Stimuli

The overall testing protocol included a novel self-report scale in trimmed form, a series of validated questionnaires and an x-ray screening task. Additional tasks were also included in the testing protocol but they were not aimed to scale validation and will thus be the object of a separate report. Only the differences from Materials used in Study 1 will be mentioned here below under the relevant headings.

###### Autism-spectrum quotient (AQ) Questionnaire and Attention to Detail Subscale

The 50-item AQ questionnaire, including the Attention to Detail subscale, was used in Phase 1.

###### Ten-Item Personality Inventory (TIPI)

The same items were used as in Study 1.

###### Novel scale: XRIndex

The XRIndex contained a selection of the six most predictive items from the proto-XRIndex pool and the four most predictive items from the Attention to Detail scale based on validation with behavioral performance in the x-ray screening task (see Study 1).

###### X-ray screening task

The same stimuli were used as in Study 1.

##### Procedure

###### Testing mode

Participants were tested online and remotely (like in Study 1) or in a laboratory. When participants underwent supervised testing in the laboratory, the software was downloaded in advance and installed on a PC by the experimenter.

###### Phase 1: web questionnaires

Phase 1 included the entire AQ questionnaire (comprising 50 items; Baron-Cohen et al., 2001), the TIPI (Gosling et al., 2003) and a 10-item version of our self-report scale (the XRIndex) which built on the evidence collected with the initial protocol. The online platform used to test participants was SurveyMonkey™.

The only procedural difference from Study 1 consisted of an additional option made available to participants at the end of Phase 1. Indeed, on the final page participants were required to select one of three options: (i) agree to proceed with the testing (and thus to be contacted via email with further instructions); (ii) withdraw from the study without allowing use of their web questionnaire data; or (iii) withdraw from the study whilst allowing inclusion of their anonymized questionnaire data in the study database.

###### Phase 2: X-ray screening task

The same procedure was adopted as in Study 1.

##### Data analysis

Data from Study 2 were used to validate the novel self-report scale with an independent sample of participants and assess possible redundancies with personality testing.

A series of Spearman’s correlations was performed to assess whether the individual characteristic measured with the XRIndex may overlap with any of the Big Five as measured with TIPI. Cronbach’s α was calculated again for the XRIndex. Five indices of performance were extracted from behavioral data in the x-ray screening task (accuracy, HFA, d’, c, RT). Performance was first explored via ANOVA or *t*-tests having Threat (threat present, threat absent) and Block (first half, second half) as within-participant factors. Linear regression models were then tested to assess the validity of XRIndex as a predictor of x-ray screening performance.

### Results

#### Non-Redundancy Between the XRIndex and the Big Five (*N* = 620)

Raw scores for each dimension measured by TIPI were calculated by reverse coding half the items and calculating the average of the two items corresponding to a dimension, as detailed in Gosling et al. (2003). Our sample of participants obtained a median (IQR) score of 5 (2) for Extraversion, 4.5 (1.5) for Agreeableness, 5 (2) for Conscientiousness, 5 (2.5) for Emotional Stability and 5.5 (1.5) for Openness to Experience. After correcting for multiple comparisons with Bonferroni-Holm (min corrected *α* = 0.005) a positive correlation between Extraversion and Emotional Stability (ρ = 0.19, *p* < 0.001), between Extraversion and Openness to Experience (ρ = 0.25, *p* < 0.001), between Agreeableness and Emotional Stability (ρ = 0.11, *p* = 0.005) and between Emotional Stability and Openness to Experience (ρ = 0.11, *p* = 0.005) were found. These correlations were all in the small/lower-medium range, as it could be expected if each of the five traits loaded on a different factor (Gosling et al., 2003).

**Figure 5:**
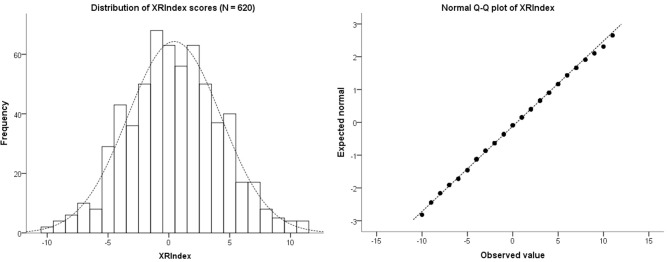
**The left hand panel shows a histogram of the distribution of scores for the XRIndex in 620 participants**. The right hand panel shows the Normal Q-Q plot of the XRIndex distribution, which in its central part essentially overlaps with the normal distribution.

The median XRIndex score was 0 (IQR = 5), and the mean was 0.45 (SD = 4). Skewness and Kurtosis of the XRIndex distribution were very close to 0 (Skewness = 0.70, SE = 0.10; Kurtosis = −0.12, SE = 0.20) and the Q-Q plot showed a reasonable fit of the data with the ideal normal distribution, especially for the central scores (see Figure 5). The XRIndex scale showed good reliability, with a Cronbach’s α of 0.70. Participants obtained a median score of 13 (IQR = 5) in the Regularities subscale and a median score of 13 in the Memory (IQR = 4) subscale. A small/moderate correlation between Regularities and Memory was found and remained significant after correction for multiple comparisons (ρ = 0.21, *p* < 0.001). In relation to personality traits, the only significant correlation between the XRIndex and the Big Five scores was a small negative correlation with Extraversion (ρ = −0.12, *p* = 0.002), which points to non-redundancy between the XRIndex and the Big Five.

#### The XRIndex as Predictor of Performance in Security X-Ray Screening (*N* = 249)

Data from the 249 participants who completed both the threat detection task and the XRIndex scale were used for behavioral validation in the UK sample. In the x-ray screening task, the same dependent variables as for the Italian sample of participants (Study 1) were considered: *accuracy* (%)*, HFA* (%), *sensitivity* [d’ = Z (hit rate)−Z (false alarm rate)], *criterion* [*c* = −0.5(Z (hit rate) + Z (false alarm rate)] and *RTs* (ms). Two main independent variables (within participants) were also included in the exploratory analysis when possible: Block (1 vs. 2) and Threat (present vs. absent).

- *Accuracy*: On average, participants responded correctly 76% (SE = 0.48) of the times and were equally accurate in the presence as in the absence of a threat (*M* = 77%, SE = 0.50 and *M* = 76%, SE = 0.60 respectively). Accuracy increased with practice within a testing session (Block 1: *M* = 74%, SE = 0.59; Block 2: *M* = 79%, SE = 0.57; *F*_(1,248)_ = 75.84, *p* < 0.001, partial η^2^ = 0.23). Practice interacted with the presence or absence of a threat, by reversing the difference between threat and no-threat trials from Block 1 to Block 2 (Block 1, threat: *M* = 75%, SE = 0.70; Block 1, no-threat: *M* = 72%, SE = 0.95; Block 2, threat: *M* = 78%, SE = 0.57; Block 2, no-threat: *M* = 80%, SE = 0.68; *F*_(1,248)_ = 14.04, *p* < 0.015, partial η^2^ = 0.05).
- *HFA*: On average participants showed an HFA difference of 56% (SE = 1.13). The percentage of HFA increased with practice, as shown by the significant difference between Block 1 and Block 2 (Block 1: *M* = 51%, SE = 1.40; Block 2: *M* = 61%, SE = 1.30; *F*_(1,248)_ = 40.76, *p* < 0.001, partial η^2^ = 0.14).
- *Sensitivity*: On average participants showed a d’ of 1.61 (SE = 0.04). Sensitivity improved with practice, as shown by the significant difference between Block 1 and Block 2 (Block 1: *M* = 1.43, SE = 0.04; Time 2: *M* = 1.80, SE = 0.05; *F*_(1,248)_ = 42.94, *p* < 0.001, partial η^2^ = 0.15).
- *Criterion*: Participants showed an average c of -0.05 (SE = 0.02). Response criterion did not change with practice (Block 1: *M* = −0.04, SE = 0.02; Block 2: *M* = −0.06, SE = 0.02; *p* > 0.24).
- *Reaction Times*: On average, participants responded correctly in 1531 ms (SE = 28) and were faster in the presence than in the absence of a threat (*M* = 1181 ms, SE = 18 and *M* = 1880 ms, SE = 40 respectively, *F*_(1,248)_ = 568.61, *p* < 0.001, partial η^2^ = 0.70). Speed increased with practice (Block 1: *M* = 1626 ms, SE = 31; Block 2: *M* = 1435 ms, SE = 27; *F*_(1,248)_ = 110.75, *p* < 0.001, partial η^2^ = 0.31) and more so for threat absent than threat present items (Threat × Block: *F*_(1,248)_ = 12.10, *p* = 0.001, partial η^2^ = 0.05).

**Figure 6:**
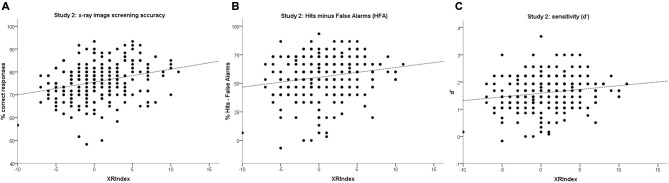
**Scatterplots with linear regression models for the XRIndex on: (A) overall accuracy (B) HFA and (C) sensitivity (d’) in Study 2**. Each point may represent one or more participants.

Linear regression models having the XRIndex as predictor revealed significant linear trends for overall detection accuracy (*R^2^* = 0.08, constant = 76, *b* = 0.56, *p* < 0.001), detection accuracy for threat present items (*R^2^* = 0.03, constant = 78, *b* = 0.43, *p* = 0.004), rejection accuracy for threat absent items (*R^2^* = 0.05, constant = 74, *b* = 0.70, *p* < 0.001), HFA (*R^2^* = 0.03, constant = 55, *b* = 0.86, *p* = 0.004) and d’ (*R^2^* = 0.03, constant = 1.59, *b* = 0.03, *p* = 0.007; see also Table 2; Figure 6) but not c or RTs (*p* = 0.108 and *p* = 0.672 respectively).

### Discussion

In Study 2 we provide a cross-cultural validation of a novel self-report scale, the XRIndex, aimed to capture baseline individual aptitudes to the security screening job with transmission x-ray images. Study 1 showed that the XRIndex, a scale which improves on Baron-Cohen et al.’s (2001) Attention to Detail scale for security x-ray screening and use in job selection settings, provides a reliable predictor of threat detection in security x-ray images. Its cross-cultural validity and robustness were then tested with an independent sample of participants from the UK. Linear regression models of XRIndex scores on screening performance fully replicated Study 1. In the new sample, the XRIndex accounted for up to 8% of the total variance across indices of performance, with a regression coefficient of 0.86 for HFA, which nicely replicates the findings of Study 1. We also showed that the source of individual variability captured with the XRIndex is not redundant with any of the Big Five traits. This non-redundancy has important practical implications, given the widespread use of personality testing in job selection settings and theoretical implications, due to the specific pattern of correlations (or lack of) between the XRIndex and the Big Five (see Wakabayashi et al., 2006 for a similar conclusion concerning the overlap between traits measured via the AQ questionnaire and the Big Five).

## Study 3

### Repetition Validity Test

#### Materials and Methods

##### Participants

Ninety-three participants (31 males) from the group who took part in the in-lab testing described in Study 2, returned on average 3 weeks later for a second testing session. Fifteen volunteers (two males) from the initial cohort appeared to drop out due to objective obstacles (e.g., overlap with job shifts or unforeseen commitments), rather than for poor motivation. The mean age of the retest sample was 25 (SD = 7) and they had spent on average 15 (SD = 4) years in education. All of them reported normal or corrected to normal vision.

##### Stimuli

The testing protocol was identical to the protocol of the first testing session (see “Study 2” Section).

##### Procedure

The same script as for the first testing session was followed (see “Study 2” Section). Whenever possible, participants were tested in the same group as at time 1 and seated at the same workstation. Same-group testing was possible for about 70% of our participants. Almost every participant could be assigned to the very same cubicle at retest, although a handful had to be moved due to rescheduling.

##### Data analysis

For the purpose of the current report, data from Study 3 were used to calculate test-retest reliability measures. The same indices of performance were extracted from behavioral data in the x-ray screening task as in previous studies. Linear regression models were tested to assess the validity of XRIndex as a predictor of x-ray screening performance at re-test.

### Results

Overall, participants showed a median XRIndex score of 0 (IQR = 5) at Time 1 and a median XRIndex of 0 (IQR = 6) at Time 2. A moderate correlation was found between individual scores obtained at Time 1 and Time 2 (ρ = 0.65, *p* < 0.001). We summarize performance outcomes for the x-ray screening task here below.

- *Accuracy*. On average, participants responded correctly 79% (SE = 0.99) of the times in the threat detection task. They were equally accurate in the presence as in the absence of a threat (*F*_(1,92)_ = 1.397, *p* = 0.240). Accuracy increased with practice within the session (Block 1: *M* = 77%, SE = 1.14; Block 2: *M* = 81%, SE = 1.04; *F*_(1,92)_ = 13.552, *p* < 0.001, partial η^2^ = 0.13). The Pearson’s correlation coefficient for overall accuracy at time 1 and time 2 was significant *r* = 0.72 (*N* = 93, *p* < 0.001).
- *HFA*. On average, participants showed a HFA difference of 60% (SE = 1.89). The percentage of HFA increased with practice, as shown by the significant difference between Block 1 and Block 2 (Block 1: *M* = 55%, SE = 2.31; Block 2: *M* = 64%, SE = 2.10; *F*_(1,92)_ = 15.59, *p* < 0.001, partial η^2^ = 0.14). The Pearson’s correlation coefficient for overall HFA at time 1 and time 2 was significant *r* = 0.61 (*N* = 93, *p* < 0.001).
- *Sensitivity*. Participants showed an average d’ of 1.82 (SE = 0.07). Sensitivity improved with practice, as shown by the significant difference between Block 1 and Block 2 (Block 1: *M* = 1.70, SE = 0.09; Block 2: *M* = 1.93, SE = 0.08; *F*_(1,92)_ = 9.90, *p* = 0.002, partial η^2^ = 0.10). The Pearson’s correlation coefficient for overall d’ at time 1 and time 2 was significant *r* = 0.62 (*N* = 93, *p* < 0.001).
- *Criterion*. Participants showed an average c of 0.01 (SE = 0.03). Response criterion did not change with practice (Block 1: *M* = 0.03, SE = 0.04; Block 2: *M* = −0.02, SE = 0.03; *p* > 0.16). The Pearson’s correlation coefficient for overall c at time 1 and time 2 was significant *r* = 0.56 (*N* = 93, *p* < 0.001).
- *RTs*. Participants responded correctly with an average latency of 1328 ms (SE = 49). They were faster in the presence than in the absence of a threat (*M* = 1031 ms, SE = 28 and *M* = 1625 ms, SE = 74 respectively; *F*_(1,92)_ = 116.10, *p* < 0.001, partial η^2^ = 0.56), which suggests a tendency to conduct exhaustive searches when no threats seem to be present. Latency improved with practice, as shown by the significant Block effect (Block 1: *M* = 1360 ms, SE = 51; Block 2: *M* = 1297 ms, SE = 47; *F*_(1,92)_ = 32.38, *p* < 0.001, partial η^2^ = 0.26). Moreover, the difference between threat and no-threat trials was accentuated by practice (Block 1, threat: *M* = 1052 ms, SE = 30; Block 1, no-threat: *M* = 1667 ms, SE = 78; Block 2, threat: *M* = 1011 ms, SE = 26; Block 2, no-threat: *M* = 1583 ms, SE = 71; *F*_(1,92)_ = 6.17, *p* < 0.015, partial η^2^ = 0.06). The Pearson’s correlation coefficient for overall RTs at time 1 and 2 was significant *r* = 0.74 (*N* = 93, *p* < 0.001).

Regression models having the XRIndex as predictor revealed significant linear trends for overall detection accuracy (*R^2^* = 0.06, constant = 79, *b* = 0.56, *p* = 0.016), detection accuracy for threat present items (*R^2^* = 0.07, constant = 80, *b* = 0.55, *p* = 0.012), HFA (*R^2^* = 0.07, constant = 60, *b* = 1.11, *p* = 0.012) and d’ (*R^2^* = 0.07, constant = 1.84, *b* = 0.05, *p* = 0.010; see also Table 2) but not rejection accuracy for threat absent items, c or RTs (*p* = 0.102, *p* = 0.801 and *p* = 0.335 respectively).

### Discussion

Repetition did not appear to undermine the validity and predictive power of the XRIndex; an important result in view of potential applications to real-world settings of the screening tool. Learning and previous exposure to the testing material do not seem to interfere with this newly described relation between a targeted self-report measure and threat detection performance with x-ray images. Because most applicants for security screening jobs will have already received professional training and obtained a competence certificate it would be most useful if our probe was expertise-proof in addition to being repetition-proof. A definitive conclusion on this point requires *ad hoc* testing with professional screeners. However, we can argue that the XRIndex may be very useful in those cases where untrained personnel require training to cover a sharp increase in the required frequency of security checks, such as with the Olympics.

## Conclusion

With Study 1, we confirmed the relation between Attention to Detail and security x-ray image interpretation skills; we also showed that the Attention to Detail scale may be deconstructed into three factors, two of which can be combined to increase the correlation between self-report and measures of performance in a prototypical x-ray screening task. Further items were developed to increase reliability of the resulting scale, which we named the XRindex. With Study 2, we validated the predictive capability of the XRIndex with a large sample of naïve participants and established its divergent validity with the Big Five personality factors. With Study 3, we tested the stability of the XRIndex score with an interval of 3 weeks on average. From a translational standpoint the XRIndex looks promising and calls for validation in the field because of its ease and the speed of its administration.

From a basic/theoretical standpoint we wonder whether other unaccounted for variables that co-vary with this measure of individual differences may account for its apparent association with x-ray screening performance. For example, it is possible that our XRIndex did not capture a “modular” trait usable to predict performance in the threat detection task with x-ray images, but rather captured a difference in general cognitive abilities or intelligence (*g*). It has previously been shown that *g* shows moderate to strong positive correlation with performance in virtually every task. Whereas broad domains of mental abilities such as reasoning, spatial ability, memory, processing speed and vocabulary explain a relatively small proportion of variance in comparison with general cognitive ability (for a review see Deary et al., 2010).

However, we are inclined to believe that the XRIndex does not act as an unspecific proxy for *g* because of the way it is calculated (i.e., by subtracting the Memory from the Regularities score). Individuals who obtain high XRIndex scores will not have rated themselves high for both the Regularities and the Memory subscale items, as would be expected if they were from the upper end of the distribution of *g* scores.

On the other hand, the Attention to Detail subscale from which the XRIndex was derived, has been shown to positively correlate with visual working memory performance. Whereas all the other AQ subscales jointly were anti-correlated with visual working memory performance in a non-clinical sample (Richmond et al., 2013). Interestingly, the correlation between Attention to Detail and visual working memory performance was specific for hit rates in a novel visual form recognition task (i.e., memory for shapes), rather than encompassing memory for the temporal order in which shapes were displayed. This converges with our finding of a strong positive relation between Attention to Detail and hit rates in Study 1. Additionally, no significant relation was found between Attention to Detail and either verbal working memory tasks (word recognition, word order memory) used in the study. Fugard et al. (2011) found the AQ Social Skill and Attention Switching scores, rather than the Attention to Detail score, to be significantly (the former positively and the latter negatively) related to overall performance in Raven’s Advanced Progressive Matrices in a student sample. Moreover, higher total AQ scores predicted better performance only for visuo-spatial and not for verbal-analytic items of the Advanced matrices. Such dissociation could not be attributed to difficulty because accuracy was equal for the two categories of items. Although no explanation is produced for the predictive value of the Social Skill score, the authors propose that poor Attention Switching may signal an executive functioning problem and thus associate with poorer ability to switch between alternative solution strategies (i.e., visuo-spatial vs. verbal-analytic) in the Advanced matrices. In people with ASD better performance on Raven’s matrices may be due to enhanced perceptual processes rather than more widespread fluid intelligence (e.g., Hayashi et al., 2008). This may also be the case for individuals with higher scores in the non-clinical portion of the distribution of autistic traits. In a recent neuroimaging study, Hao et al. (2013) isolated specific anatomo-functional correlates of individual differences in performance at the Embedded Figure Test (a task of visual field independence and piecemeal attention; Shah and Frith, 1983) after controlling for the effects of fluid intelligence, as measured with the Raven’s Progressive matrices, age and sex. Overall, this suggests that whilst classical intelligence tests may tap on multiple cognitive components, some of which are relevant for attention to detail, the latter rests on a more restricted set of skills some of which are more basic and encapsulated than general intelligence measures. Lastly, only small positive correlations (with rs equal to 0.09 and 0.15) were reported between performance in Raven’s Advanced Matrices and performance in the X-Ray ORT (a threat detection test with very similar rationale as our x-ray probe test) in two large samples of x-ray screeners under training (Hardmeier and Schwaninger, 2008).

An interesting finding of the present study was that the predictive value of the Attention to Detail scale does not rest with items with a strong emphasis on detail focus or long-term memory. The latter in particular were anti-correlated with x-ray screening performance. Although these negative correlations were small and failed to reach significance after correction for multiple tests, we found that subtracting the Memory score from the combined score for Regularities enhanced the predictive power of the original Attention to Detail items. This would seem counterintuitive, given the obvious relevance of memory processes for the recognition of known threats in security x-ray images (Schwaninger, 2006). However, the Attention to Detail items focusing on memory are essentially concerned with long-term memory for symbolic material—more precisely, phone numbers and dates of birth—and such form of memory may rely on distinct neural substrates than long-term memory for visual objects (Denes and Pizzamiglio, 1998). On the other hand, we also speculate that having an eidetic-like memory might interfere with, rather than facilitate (Luria, 1988), ongoing processing and the interpretation of novel stimuli like security x-ray images, and consequently hamper screening performance. The tradeoff between over-reliance on long-term memory detail and focus on online pattern recognition is what we may be capturing with the XRIndex.

The ambiguity of the XRIndex scale, whose total score (unlike the Attention to Detail score) is derived by subtracting the scores obtained in two subscales, Regularities and Memory, is a potential issue from a theoretical standpoint. Indeed, this characteristic implies that individuals with the highest XRIndex scores will have obtained top scores for Regularities and bottom scores for Memory. While individuals with the lowest XRIndex scores will have obtained bottom scores for Regularities and top scores for Memory. Individuals scoring around 0 will have similar scores for Regularities and Memory, and these could be either both high or both low. For both Regularities and Memory the autistic option received the highest score, thus individuals receiving top scores in the XRIndex score would not necessarily receive top scores in the Attention to Detail subscale of the AQ questionnaire (as also suggested by the small positive correlation found between the two scales; see Study 1, Result section).

In order to obtain top scores in the Attention to Detail scale, individuals require high scores on the four Attention to Detail items (included in the XRIndex) loading on Regularities and Memory, but also on the two other items loading on Detail and on the four remaining Attention to Detail items with weaker loadings overall. This suggests that it is not possible to draw simplistic predictions about individuals with high Attention to Detail score falling around the middle range (i.e., centered on 0) of the XRIndex. Furthermore, empirical data used in the selection of Attention to Detail components to be included in the XRIndex also suggest that the difference between Regularities and Memory—instead of particularly high scores for Regularities or particularly low scores for Memory alone—provides the strongest predictor for performance in x-ray screening (see Study 1, Result section). Although the utility criterion (or finding “what works”) may be sufficient justification for the existence and use of a new scale in an applicative context, it would be interesting to further investigate the conceptual basis of the specificity of the XRIndex (i.e., to understand “why it works”), paving the way for further practical improvements but also theoretical advances.

Because the XRIndex is a short self-report scale intended for use in security personnel selection settings, its opacity to interpretation represents an advantage in applicative terms. If prospective employees were handed the Attention to Detail scale, they would easily hypothesize that the options showing enhanced analytical skills and eye for detail may look more desirable. Thus, the total Attention to Detail score could be more vulnerable to manipulation by strategic choices. If prospective employees were handed the XRIndex scale, they would still hypothesize that the options showing enhanced analytical skills and eye for detail may look more desirable. However, they would probably not be able to guess that the total score is calculated by subtracting the score from two subscales (both including a similar number of reverse-coded items and positive/negative sentences), and that endorsing the analytical style in all of the items will actually place them around the middle range of the score distribution.

Although this is a positive aspect of the tool we developed, it still does not guarantee that every individual is capable of reporting their characteristics in a way that accurately represents reality, even in the absence of any intentional strategies. For this reason, the tool may become more reliable and acquire more predictive power if it was also available in a version for external observers who are well acquainted with the individual, to confirm or adjust the score obtained via self-reports.

Finally, while the 50–50 split between threat-present and threat-absent images, which we adopted in the x-ray screening task, may be ideal for the large scale testing of naïve participants, it is not representative of actual proportions of true/projected threats on the job. However, this does not detract from the fact that, all things being equal, the XRIndex is related to detection performance. Wolfe and van Wert (2010) and Wolfe et al. (2013) suggest that the effects of target prevalence in two-choice x-ray screening tasks may be due to different mechanisms than in vigilance tasks (Davies and Parasuraman, 1982). In particular, target prevalence was shown to affect response criterion (c) and response speed for target absent trials. The question is: will target prelavence interact with individual differences as captured by the XRIndex in a way that undermines its utility? Although it is primarily an empirical question, we speculate that this is unlikely to be the case as the variance captured by the XRIndex is related to d’ rather than c or response speed, and whereas c and response speed appear to be affected by target prevalence, d’ does not.

In summary, the XRIndex could be useful in a scenario where large samples of non-professionals need to be recruited for the screening job at major mass events. The London 2012 Olympic and Paralympic Games provided a clear example in which individuals had to be recruited on an *ad hoc* basis in the absence of previous training (London 2012 Olympic and Paralympic Safety and Security Strategy, 2011). The use of a short scale would help identify individuals who may be predisposed to the screening task, due to perceptual abilities enabling them to tackle image-based challenges, such as object superposition, better than an average observer. The ability to tackle some of these challenges, indeed, cannot be easily improved by training or job-specific experience (Schwaninger et al., 2005; Hardmeier and Schwaninger, 2008).

## Conflict of Interest Statement

The authors declare that the research was conducted in the absence of any commercial or financial relationships that could be construed as a potential conflict of interest.
